# Quantification of the magnification and distortion effects of a pediatric flexible video-bronchoscope

**DOI:** 10.1186/1465-9921-6-16

**Published:** 2005-02-10

**Authors:** IB Masters, MM Eastburn, PW Francis, R Wootton, PV Zimmerman, RS Ware, AB Chang

**Affiliations:** 1 School of Medicine, Discipline of Paediatric and Child Health, University of Queensland, Herston 4029, Brisbane, Australia; 2 University of Queensland, Department of Information Technology and Electrical Engineering, St Lucia 4072, Brisbane, Australia; 3 Department of Respiratory Medicine, Royal Children's Hospital, Herston 4029, Brisbane, Australia; 4 University of Queensland Centre for Online Health, Level 3 Foundation Building, Royal Children's Hospital, Herston 4029, Brisbane, Australia; 5 Department of Thoracic Medicine, The Prince Charles Hospital, Rode Rd, Chermside 4032, Brisbane, Australia; 6 Longitudinal Studies Unit, School of Population Health, The University of Queensland, Herston 4006, Brisbane, Australia

**Keywords:** Flexible bronchoscopy, Pediatric, Magnification, Distortion

## Abstract

**Background:**

Flexible video bronchoscopes, in particular the Olympus BF Type 3C160, are commonly used in pediatric respiratory medicine. There is no data on the magnification and distortion effects of these bronchoscopes yet important clinical decisions are made from the images. The aim of this study was to systematically describe the magnification and distortion of flexible bronchoscope images taken at various distances from the object.

**Methods:**

Using images of known objects and processing these by digital video and computer programs both magnification and distortion scales were derived.

**Results:**

Magnification changes as a linear function between 100 mm (×1) and 10 mm (×9.55) and then as an exponential function between 10 mm and 3 mm (×40) from the object. Magnification depends on the axis of orientation of the object to the optic axis or geometrical axis of the bronchoscope. Magnification also varies across the field of view with the central magnification being 39% greater than at the periphery of the field of view at 15 mm from the object. However, in the paediatric situation the diameter of the orifices is usually less than 10 mm and thus this limits the exposure to these peripheral limits of magnification reduction. Intraclass correlations for measurements and repeatability studies between instruments are very high, r = 0.96. Distortion occurs as both barrel and geometric types but both types are heterogeneous across the field of view. Distortion of geometric type ranges up to 30% at 3 mm from the object but may be as low as 5% depending on the position of the object in relation to the optic axis.

**Conclusion:**

We conclude that the optimal working distance range is between 40 and 10 mm from the object. However the clinician should be cognisant of both variations in magnification and distortion in clinical judgements.

## Introduction

The flexible bronchoscope has been used in pediatrics for more than 20 years [1] yet there are only a limited number of publications on the systematic examination of the physical properties of magnification and distortion found in endoscopes of any size, let alone bronchoscopes specific for the pediatric sized airways. [1-6]. Some understanding of specific bronchoscopic magnification and distortion is an important issue to the clinician as these instruments are being used more regularly to define the nature and severity of airway lesions such as tracheal stenosis and malacia disorders from which important medical and surgical decisions ultimately follow[7-14]. The pediatric bronchoscope comes in a variety of sizes but the resultant magnification from these instruments depends not only on the size but also on the image processing that occurs.

Magnification of an object refers to the virtual or real visualized and measured increase in size of that object after rays of light from that object have passed through a lens system. Magnification varies across the field of view and with the distance of the lens from the object [15]. Distortion refers to the variations of the faithful representation in scale and perspective of the various elements of the object and its plane of context[15]. There are various forms of distortion including geometrical, curvilinear, anamorphic and perspective distortion recognised in photometrics [15]. Geometrical distortion refers to the changes in the peripheral details because of elongation of the elements of an object. Simply, it refers to the distortion that is created when there is increasing obliquity of the angle of viewing of the object. Curvilinear distortion is described where straight lines are rendered as curved either inward (pincushion) or outwards (barrel) curves. This form of distortion is a result of asymmetry of lens configuration and is a feature of bronchoscopes and indeed most endoscopes in general[2,5,16]. The ultimate image quality of any bronchoscope is dependent on the lens characteristics in general and the subsequent transmission of the image to the image processor and display module. The more recently developed bronchoscopes such as the flexible videobronchoscope (FVB's) have moved away from fibreoptics to transmit images. They use charge coupled devices (CCD's) which are mounted adjacent to the lens thus essentially converting the image to electrical energy at the lens. This early conversion of the image has the effect of producing "less noise" thus allowing greater image clarity. Despite the latter, all of these issues mentioned are important to the clinical use of FVB's and subsequent patient management and as the Olympus company was unable to provide the necessary details on magnification and distortion, we undertook to quantify these effects in order to enhance our decision making quality during FVB procedures. The aim of this paper is to define the magnification and distortion in a commonly used pediatric video bronchoscope: the Olympus BF Type 3C160.

## Methods

### Equipment

A flexible bronchoscope (Olympus BF Type 3C160, Olympus, Tokyo, Japan) and light source (Olympus Evis Exera CLV-160, Olympus, Tokyo, Japan) and processor (Olympus Evis Exera CV-160, Olympus, Tokyo, Japan) were connected to a TV monitor (Sony HR Trinitron, Sony Corporation, Shinagawa-ku, Tokyo, Japan) and a colour video printer (Sony Mavigraph, Sony Corporation, Shinagawa-ku, Tokyo, Japan) as supplied by the Olympus company. The image signals from the Evis Exera CV-160 were co-recorded by a digital camera (Sony Mini DV Digital Handycam, Sony Corporation, Shinagawa-ku, Tokyo, Japan). These digital video images were viewed and stored as uncompressed 640 × 480 pixel, millions of colours as TIFF files. These images were then analysed by an image processing program (Image J, Wayne Rasband, National Institute of Health, USA) and a computer (MAC Power Book G4. Apple Inc,, Cupertino, Ca., USA).

### Measurement of Magnification and Distortion

**Figure 1:**
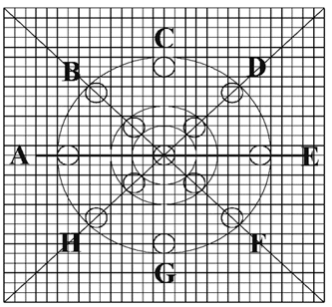
Photographic Object: A square of graph paper 3 cms × 3 cms with 1 mm × 1 mm units and concentric circles of varying diameter (2 mm, 6 mm, 10 mm and 20 mm) and diameter markings (AE, BF, CG, DH) all generated by Auto CAD.

**Figure 2:**
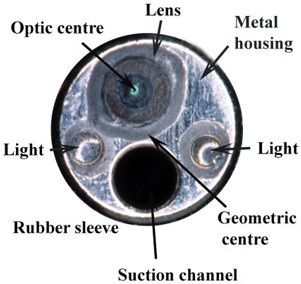
The end view of the tip of the Olympus BF Type 3160 bronchoscope displaying the "off set" lens and lights and the geometric centre.

A precisely measured object consisting of 4 concentric circles with emanating radii on 1 mm × 1 mm graph paper was created and drawn to precision using design software (Auto CAD, Autodesk, San Rafael, Ca, USA) (Fig 1). These circles were 2, 6, 10 and 20 mm diameter respectively. A precision made device that fixed the hand piece and supported the length of the bronchoscope so that the central or geometric axis of the bronchoscope (Fig. 2) would align with the centre point of a moveable screen was engineered by Queensland Radium Institute (QRI, Brisbane, Australia) engineering. The device's moveable stage allowed the object to be positioned over a range of distances from 100 mm to 3 mm without moving the scope itself. The measured distances were verified with electronic callipers to a degree of accuracy of 0.02 mm. The calibration process for calculation of magnification and distortion was performed at the distance of 100 mm, the point at which the object to image ratio was 1. All subsequent ratios were therefore larger than the calibration value and represented the magnification of the object.

**Figure 3:**
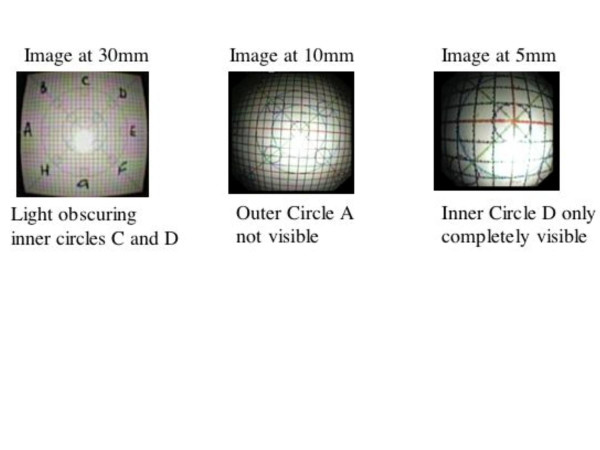
Effects of distance from object on the image appearance within the Field of View (FOV) and Light intensity obscuring parts of the image. Note the barrel appearance of the graph paper at the periphery in image "A" while in image "C" these effects are clearly offset or asymmetrical.

Magnification was calculated by repeatedly measuring the image diameters AE, BF, CG, DH across the 4 concentric circles (Fig. 1) at distances of 100, 80, 60, 40, 30, 25, 20, 15, 10, 5 and 3 mm and then dividing these measurements by the corresponding value of the object. These magnification measurements were repeated in 3 separate experiments and the mean value for all of the diameters of that particular circle at that particular distance was taken as the final magnification for that circle at that distance. Between the distances of 100 mm and 40 mm the calculations of the inner circle were incomplete or impossible because of the effects of the light spot obscuring the object (Fig. 3a). Similarly as the lens approached very close to the object (5 mm to 3 mm) the outer circles became incomplete or outside the field of view (Fig. 3b).

Geometrical distortion was defined as the radial length ratios from each interlocking circle with radii A taken as the denominator for all subsequent radial measurements within the outer circle and then other elements of radius A for the other circles. The uniformity of the ratios was then tested in quadrant or sectors: a sector or quadrant being an area defined in accordance with mathematical convention[17] whereby sector 1 is the NNE sector and sector 2 is NNW continuing in a anticlockwise direction for sectors 3 and 4. The comparisons were as follows: sector 1 with sector 3; sector 2 with sector 4 or the diagonal sectors with their respective opposites. This provided an understanding of the homogeneity of the distortion profile or geometrical distortion and also gives reference to curvilinear distortion.

The changes in magnification across the field of view was further defined as the differences in calculated measurements for a known distance of 2 mm across the field of view at a fixed distance (15 mm) from the object. These measurements were made in the horizontal plane. As with the magnification calculations, the mean distortion value from the entire set of 3 experiments was taken as the defined distortion at the particular distance from which the measurements were made. All of the aforementioned experiments were then repeated on 3 separate Olympus BF Type 3C160 bronchoscopes. The experiments were performed separately with the central geometrical axis of the bronchoscope and then the optic axis of the bronchoscope (Fig. 2) aligned with the centre point of the stage and object.

### Statistics

Magnification and distortion were expressed as mean values and comparisons between geometric and optical axis measurements were assessed by the Student's t test. A repeat measures ANOVA was used to assess the effects of distance, circle and sectorial effects. The reliability of the repeated measurements of magnification between tests and between bronchoscopes was assessed by intra-class correlation. Data storage and statistical calculations were performed on SPSS for MAC version11 (SPSS Inc., Chicago, Il, USA).

## Results

**Figure 4:**
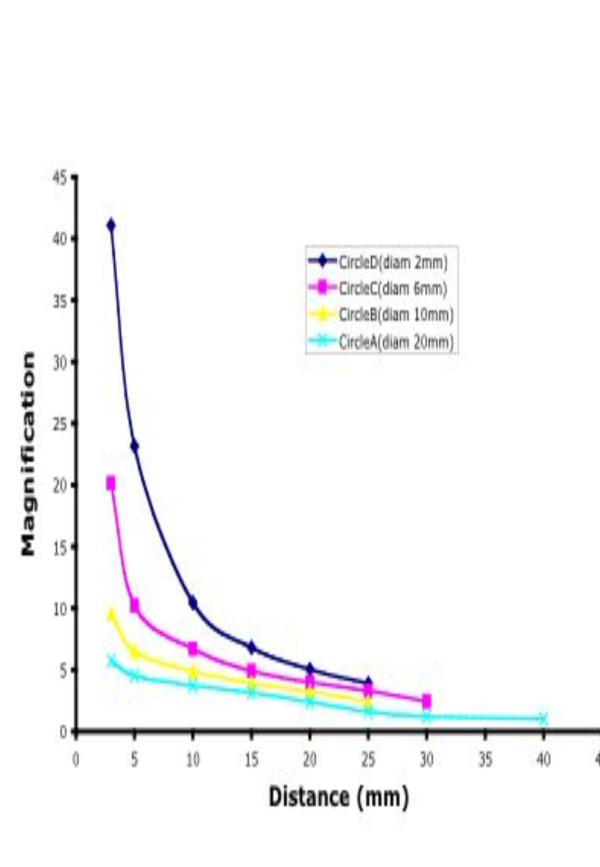
Effects of position within the Field of View: Magnification changes from 40 mm to 3 mm from the object displaying the variable exponential changes in magnification close to the object.

The mean linear magnification for the bronchoscopes aligned along the central geometrical axis of the bronchoscope over the range of the depth of field from 100 mm to 3 mm is shown in Additional file 1. The magnification progressively increased in a linear fashion between 100 and 10 mm from the object and then exponentially increased to 40 times between the distances of 10 mm and 3 mm from the object. The magnification factor was approximately ×10 at 10 mm and ×40 at 3 mm from the object. The graphic representation of these values as tightly fitting linear becoming exponential curves is displayed in Figure 4. The goodness of fit (R^2 ^value) for each of the curves for circles A,B,C,D was 0.999, 0.999, 0.999 and 0.999 respectively. When the bronchoscope was aligned to the object through the optical axis, the magnification was reduced generally but by as much as 22% at 5 mm and 14.5% at 10 mm from the object (Additional file 2).

The mean intra-class correlation alpha level for the repeated measurement of magnification ranged from 0.9369 (95%CI: 0.6167 to 0.9878) to 0.9811 (95%CI: 0.8827 to 1.000). These intraclass correlation data indicate and support the goodness of fit of the regression curves. The variability in magnification measurement between the bronchoscopes was extremely small and there are virtually no differences between the bronchoscopes (Table 1). The reliability coefficient average alpha value for the measurements from 3 separate bronchoscopes assessed at 10 mm was 0.9996 (95%CI: 0.9991 to 0.9999).

**Table 1:** Inter-scope comparison of magnification with optical axis aligned.

**Bronchoscope No.**	**Distance mm**	**Circle A**	**Circle B**	**Circle C**	**Circle D**
324	30	3.0938	3.1601	3.2913	
292	30	3.0984	3.1963	3.3258	
039	30	3.0820	3.2083	3.3191	
					
324	15	5.5356	6.2137	6.4443	6.4918
292	15	5.5016	6.1527	6.3062	6.3832
039	15	5.4997	6.1107	6.6364	6.4358
					
324	10		8.9428	9.5024	9.6755
292	10		8.5663	9.0650	9.3453
039	10		8.5663	9.0649	9.3449
					
324	5			17.6819	19.8996
292	5			15.9314	17.0974
039	5			15.6890	16.7272
					
324	3				32.4274
292	3				26.1051
039	3				24.7775

**Figure 5:**
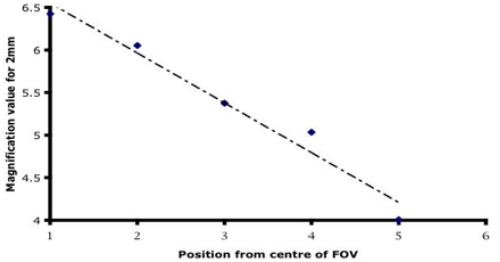
Across lens magnification: the near linear changes in magnification across the horizontal plane from the centre to periphery of the lens.

**Figure 6:**
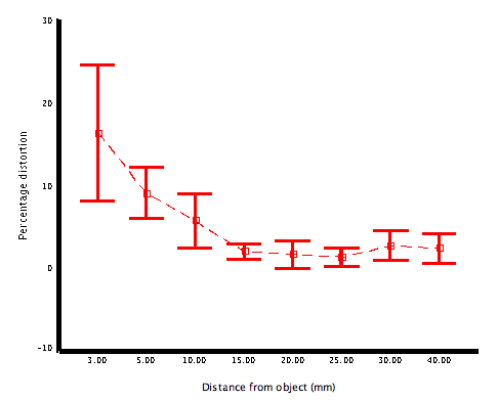
Mean central distortion ± 95% CI for the central or geometrical axis aligned bronchoscope.

The across field magnification was greatest in the centre of the field and least at the periphery with some 38.5% reduction in magnification across a 20 mm diameter object but only 15.4% across an object of 6 mm diameter (Figure 5). The overall distortion including geometric distortion for the bronchoscopes aligned along the central axis ranged from near zero at 40 mm, 5% at 5 mm but at 3 mm it had risen to 30% (Figure 6). When the object very closely approximated the optical axis in alignment (object centre is within 2 mm of the lens's optical axis), the overall magnification factors changed (see additional file 2) but importantly this value of distortion at 3 mm from the object was markedly reduced to 5%. Distortion was significantly different in different quadrants when the scopes were aligned along their central geometrical axes to the object.

The appearance of the curvilinear distortion is shown in Figure 3 and clearly displays barrel distortion. The measurements show that curvilinear distortion was different for the different parts of the field of view. In particular, the magnification at the centre of the field of view at any distance was different to the periphery (Figure 5). These differences were not evenly distributed across the field of view, however the differences between quadrants were extremely small. A univariate analysis found significant effects for ring, distance and sector analysis. However the three factor repeat measures ANOVA analysis revealed no significant differences between measures by sector (adjusted p-value using Box's conservative test = 0.13). When the data was stratified by distance, all 8 p-values were not significant (0.13 to 0.31), indicating that a mild effect by sector may exist.

## Discussion

This is the first reported study detailing systematically the magnification and distortion factors that surround the optic properties of a commonly used pediatric video-bronchoscope. The magnification progressively increases in a linear fashion over the distances of 100 mm to 10 mm with changes from 1 to near 10 fold magnification. However below 10 mm, the magnification changes exponentially thus making appreciation of the actual or real size of the object very difficult even when the distance from the object is known [3,5,6]. This study also shows that the magnification of the instrument changes is accordance with the axis of orientation of the bronchoscope to the object. These data indicate that the optimal operating distance is between 40 mm and 10 mm where the mean magnification is linear and is 3 to 9.5 fold and the distortion of any form can be under 5%. However it also means that it is important for the bronchoscopist to maintain a "perspective of operating distance" or distance from the object when judgements of size are to be made. This study has also shown that the Image J and BTV programs and MAC power book G4 can be readily interfaced with Olympus BF Type 3C160 to produce an objective measurement package. In addition this system could be readily adapted to most image acquisition systems and varieties of flexible bronchoscopes types (FVB and fibre optic) that must be in clinical use around the world.

With respect to distortion, this study has shown that geometric distortion can result with very significant errors in size perception with up to 30 percent distortion occurring if the lens is placed very close to the object (<5 mm) and the object is not aligned through the optical axis. This could be corrected by movement of the object closer to the line of the optical axis of the lens. However there are no markers on the bronchoscope to indicate that this position has been gained. At close proximity to this point there is separation of the light beams, however while the use of this is possible in laboratory settings, it is not currently possible in the clinical context where there are differences in reflectance and absorption of light. The second issue is that barrel distortion is generally regarded as homogeneous and independent of working distance however when the lens is offset as it is in this type of instrument it cannot be, given the differences between the geometrical and optical axes of the bronchoscope. In the clinical context these issues indicate that clinicians must be aware that their judgements always contain "distortion effects" but particularly when working very close to the object eventhough the image appears clear and is centred on the screen.

The limitations of this study are the fact that the object positioning could only be moved with micrometer precision in the longitudinal plane. However an instrument that allowed for lateral movement of the object with micrometer precision may have produced less distortion but in the clinical context such precision could only ever be transient and thus these methods have allowed for a greater appreciation of the extent and complexity of distortion. Even though computer based systems have been used to correct for some of the elements of distortion[3,5,6] none exist in routine use within the current hardware of the bronchoscopes themselves. In those that have been described [5], it is unclear as to what type of distortion has been corrected. These authors have aligned their image centre or Field of View (FOV) as the centre for their measurements. However as shown in this study, correction for the offset position of the lens is important to any real measurement when the object is closer than 5 mm. If this is not done, then correction formulations will overestimate the levels of distortion in some instances, as there would be a high likelihood that the objects of view would pass through all axes of orientation during an in vivo procedure. Despite these arguments, the overall distortion of any type is extremely low and in the clinical context these values are highly acceptable as far as measurements are concerned. Also it is unlikely that an object will be viewed at this distance for any period of time given the "blurring effects" of the distortion. However during passage of the bronchoscope across lesions, clearly such transients in magnification and distortion could be misleading.

The clinical relevance of this work may not be obvious. However, we argue that it has a number of important clinical applications and that all bronchoscopists should be cognisant of these factors, which may influence their judgement of the size of lesions. Firstly, the distance of the tip of the scope to the lesions being assessed must be measured and not approximated by judgement as distances under 5 mm may result in very considerable differences in magnification and thus the estimates of size or even presence or absence of a lesion. The most obvious example of this is in assessments of a curvaceous left main stem bronchus that may initially appear as malacia but appears to become "normal" as it is viewed at a closer distance. Indeed in defining malacia a viewing or operating distance must be stated otherwise these perspective difficulties will prevail and comparative statements and judgements become meaningless. Another example occurs when assessing malacia or airway cross sectional area changes across the respiratory cycle. In that scenario the movement of the object is away from the lens during inspiration making the image relatively smaller than is real for the increase in lung volume. These optic effects combined with the venturi effects from the bronchoscope itself on reducing intraluminal pressure again compound the issue and reduce the image size or its relative changes. Bronchoscopists need to be aware of these apparent paradoxical effects, that the lumen is greatest at the end of expiration. Indeed at this point in time and until the distance of airway movement can be measured in vivo, measurements can only be made precisely at one point in the respiratory cycle, and that generally is the end expiratory point. If end inspiration is used there is likely to be intrusion of the mucosa into the field of view during the subsequent expiration. The most recent published example of most of these effects can be seen in the images provided in that paper by Okasaki et al [18] where the instrumentation magnification is not known, the measurements are not calibrated at a defined time in the respiratory cycle, the viewing distance is not described and there is clearly image change across the induced respiratory cycle.

Secondly, paradoxical concepts also apply to the use of the flexible bronchoscope in assessment and or removal of foreign bodies situated peripherally in the airways. When dealing with peripheral or markedly angulated bronchial branches it is not always possible to position the image in the centre of the screen. Here estimates of size may be misconstrued by the interactions of variable magnification across the lens, curvilinear distortion and potentially geometric distortion. These effects and the "iceberg effects" of the presented foreign body size usually mean that the foreign body appears smaller than it really is and the inexperienced bronchoscopists might tend to dismiss the object as trivial or inconsequential. To the contrary attempts to remove it should be maximized. Finally, in terms of research, defining the position of the bronchoscope in terms of the lesion being assessed and photographed is vital if realistic comparisons are to be made across time or between groups. In this regard techniques that allow for distance and lesions measurement and assessment need to be developed and integrated into routine clinical use. Using our technique described in this paper, we are quantifying airway size in a variety of airway lesions (eg tracheobronchomalacia) associated with significant respiratory morbidity.

The fact that performance of each bronchoscope was remarkably similar in terms of magnification and distortion measurements obviously reflects company's production quality. In a clinical session where a number of bronchoscopes might be used and in research where inter-group comparisons might be desired, this information suggests that a significant level of confidence can be maintained in perception terms for the bronchoscopists. This does not negate the need for individual bronchoscope calibration and for objective and accurate measurements in bronchoscopic work. Detailed data from the Olympus Company was not available for comparison; however our own validation experiments show these data to be correct and accurate. Despite the latter, as there are now many instruments available to the clinician, we suggest that companies and manufacturing regulators make readily available the magnification and distortion characteristics of each instrument type or size so that more effective clinical appraisals can occur.

Although this study has shown that the Olympus BF Type 3C160 video-bronchoscopes produce remarkably consistent magnification across the working ranges of 100 mm to 3 mm from the object, an understanding of the influence of distance on magnification and distortion of the image obtained by a flexible bronchoscope is an essential step in the development of an invivo technique of measuring airway sizes. We have provided graphic appreciation of these effects and the importance of optical versus geometric axis orientation. The optimal working distances for this bronchoscope are between 40 mm and 10 mm from the object. The study also provides reasonable working magnification factors for this type of bronchoscope and as such could allow for a better appreciation of real or actual airway sizes.

## Supplementary Material

Additional File 1 Mean magnification and the mean whole of field magnification at defined distances. Click here for file

Additional File 2 Comparison of bronchoscope axis and optical axis aligned magnification measurements. Click here for file
