# Supplementary material for: Quantification of the magnification and distortion effects of a pediatric flexible video-bronchoscope
Source: Respir Res. 2005 Feb 10;6(1):16. doi: 10.1186/1465-9921-6-16 (PMC549513; doi:10.1186/1465-9921-6-16)
Supplement: Additional File 1 — Mean magnification and the mean whole of field magnification at defined distances. [file 1465-9921-6-16-S1.doc]

**Additional file 1:** Mean magnification and the mean whole of field magnification at defined distances

| **Magnification Power** | | | | | | | | | | | |
| --- | --- | --- | --- | --- | --- | --- | --- | --- | --- | --- | --- |
| Distance mm | 100 | 80 | 60 | 40 | 30 | 25 | 20 | 15 | 10 | 5 | 3 |
| Circle A | 0.9930 | 1.2060 | 1.5966 | 2.3819 | 3.1501 | 3.7264 | 4.5264 | 5.7364 |  |  |  |
| Circle B |  |  |  | 2.4193 | 3.2438 | 3.9110 | 4.8572 | 6.5088 | 9.5366 |  |  |
| Circle C |  |  |  | 2.4321 | 3.2908 | 3.9765 | 4.8786 | 6.6851 | 10.204 | 19.966 |  |
| Circle D |  |  |  |  |  |  | 5.0294 | 6.7938 | 10.459 | 23.164 | 41.096 |
| **Mean whole of field magnification** | | | | **2.382** | **3.1785** | **3.8736** | **4.7289** | **6.2532** | **9.5519** | **21.902** | **38.124** |
