# Supplementary material for: Quantification of the magnification and distortion effects of a pediatric flexible video-bronchoscope
Source: Respir Res. 2005 Feb 10;6(1):16. doi: 10.1186/1465-9921-6-16 (PMC549513; doi:10.1186/1465-9921-6-16)
Supplement: Additional File 2 — Comparison of bronchoscope axis and optical axis aligned magnification measurements. [file 1465-9921-6-16-S2.doc]

**Additional file 2.** Comparison of bronchoscope axis and optical axis aligned magnification measurements.

| **Distance**  **mm** | **Circle** | **Scope Axis Aligned** | **Optical Axis Aligned** | **P Value** | **95%CI (low)** | **95%CI(high)** |
| --- | --- | --- | --- | --- | --- | --- |
| 40 | A | 2.3819 | 2.3277 | 0.054 | -0.0565 | 0.1646 |
| 30 | A | 3.1501 | 2.9650 | 0.021 | 0.06868 | 0.30158 |
| 30 | B | 3.2438 | 3.0510 | 0.002 | 0.15157 | 0.23393 |
| 30 | C | 3.2908 | 3.0468 | 0.004 | 0.22566 | 0.26234 |
| 20 | A | 4.5264 | 4.2621 | 0.020 | 0.9969 | 0.42891 |
| 20 | B | 4.8572 | 4.5061 | 0.002 | 0.28102 | 0.42122 |
| 20 | C | 4.8786 | 4.6339 | 0.119 | -0.15544 | 0.64491 |
| 20 | D | 5.0294 | 4.6144 | 0.104 | -0.45156 | 1.28156 |
| 15 | A | 5.7364 | 5.2395 | 0.002 | 0.39625 | 0.59740 |
| 15 | B | 6.5088 | 5.8364 | 0.004 | 0.48026 | 0.86460 |
| 15 | C | 6.6851 | 6.0673 | 0.006 | 0.41295 | 0.82274 |
| 15 | D | 6.7938 | 6.0711 | 0.057 | -0.07891 | 2.19441 |
| 10 | B | 9.5366 | 8.4476 | 0.011 | 0.86072 | 1.31738 |
| 10 | C | 10.2038 | 8.9905 | 0.020 | 0.72724 | 1.69926 |
| 10 | D | 10.4594 | 8.9416 | 0.044 | 0.18353 | 2.85197 |
| 5 | C | 19.9658 | 16.4163 |  |  |  |
| 5 | D | 23.1642 | 18.1157 |  |  |  |
